# Hydrosols of *Veronica* Species—Natural Source of Free Volatile Compounds with Potential Pharmacological Interest

**DOI:** 10.3390/ph15111378

**Published:** 2022-11-09

**Authors:** Marija Nazlić, Karla Akrap, Dario Kremer, Valerija Dunkić

**Affiliations:** 1 Faculty of Science, University of Split, Ruđera Boškovića 33, HR-21000 Split, Croatia; 2 Faculty of Pharmacy and Biochemistry, University of Zagreb, A. Kovačića 1, HR-10000 Zagreb, Croatia

**Keywords:** benzene acetaldehyde, gas chromatography-mass spectrometry, hydrodistillation, microwave extraction, *β*-ionone, antioxidant activity, ORAC, DPPH

## Abstract

In this study, free volatile compounds (FVCs) were isolated from the water fractions (hydrosols) of 10 Croatian *Veronica* species obtained by hydrodistillation (HD) and microwave-assisted extraction (MAE). The use of different isolation techniques is important for the analysis of the influence of extraction conditions on the qualitative and quantitative composition of the isolated constituents. The composition of the hydrosols was analyzed using gas chromatography and mass spectrometry. The compounds *β*-ionone and benzene acetaldehyde were detected in all 10 *Veronica* hydrosols studied. *E*-caryophyllene was also identified in all isolates except the MAE isolate of *V. arvensis* L. Caryophyllene oxide was isolated in all isolates apart from the HD isolate of *V. catenata* Pennell. (*E*)-*β*-Damascenone is significantly present in all isolates except the MAE isolates of *V. catanata* and *V. cymbalaria* Bodard. In these two species, *α*-muurolol was identified in a high percentage. The same basic phytochemical constituents and compounds characteristic of a given *Veronica* species suggest the importance of further research. Antioxidant activity was tested for all extracts using two methods, ORAC and DPPH. Therefore, it is crucial to identify as many specialized metabolites from *Veronica* species as possible, especially hydrosols, which are natural products of potential pharmacological interest.

## 1. Introduction

Specialized metabolites or secondary metabolites are natural plant products, and hundreds of compounds have been isolated and identified from all plant species studied [1]. It is precisely because of the diversity and bioactivity of these metabolites that people have used plants in traditional medicine since ancient times. The identification and mode of action of natural products isolated from different plants remains a challenge for phytochemistry and pharmacology. The importance of research on plant specialized metabolites is demonstrated by the fact that several traditional herbal medicines have been developed and clinically tested in Europe and have found their way into modern registered medicine [2]. Therefore, further systematic research of specialized plant metabolites is of great importance.

The representatives of 62 *Veronica* species (family Plantaginaceae) are widely distributed throughout Europe and their diverse uses in traditional medicine are well known [3]. Previous scientific research on the *Veronica* species has focused on iridoid glycosides and phenolic components, which constitute an important part of the specialized metabolites. Nearly 300 natural compounds of *Veronica* species have been isolated and identified and their biological activity has been studied [4,5]. Although this represents a large number of identified natural compounds in the *Veronica* species, many specialized metabolites have not yet been identified. For this reason, a systematic study of the specialized metabolites that make up the free volatile compounds (FVCs) is being conducted. Dunkić et al. [6] investigated the composition of FVCs of the 21 Croatian *Veronica* species contained in the lipophilic layer of extracts obtained by classical (hydrodistillation, HD) and green (microwave-assisted, MAE) extraction [6]. These extraction methods yield the two layers mentioned earlier, the lipophilic layer (LL) or essential oil (EO) and the aqueous layer (hydrosols). Many compounds such as hydrophobic hydrocarbons may be present in the lipophilic phase, so the composition of lipophilic and hydrosol phase isolates may differ significantly [7]. Hydrosols are aromatized waters produced by the condensation of water vapor in distillation processes. They contain a smaller amount of volatiles, which are water-soluble components and are therefore safer for human use compared to components from more concentrated lipophilic phase [8,9]. In general, research on the aroma profiles of hydrosols and their biological efficacy is still limited [10,11,12].

The composition of the volatile components of hydrosols is the subject of the present study. Ten Croatian *Veronica* species were selected for this study according to the flowering calendar. The timing of flowering is important because one of the determining factors is the appearance of the flower or inflorescence. In addition, the production of bioactive compounds is very intensive in the blooming period. Some *Veronica* species begin flowering in Croatia during January or February, such as *V. cymbalaria* Bodard, *V. arvensis* L., and *V. persica* Poir. Some of them, such as *V. persica*, can be seen blooming throughout the year. Most species bloom from March to July (*V. anagallis-aquatica* L., *V. anagalloides* Guss., *V. polita* Fr.). Thus, species from genus *Veronica* are available for research almost throughout the year. The availability of fresh plant material contributes to the quality and stability of the isolates in the lipophilic and especially in the hydrosol phase.

The first part of this research focuses on the identification of compounds in the hydrosol composition of selected *Veronica* taxa using gas chromatography-mass spectrometry (GC-MS). Therefore, another important part of this research is focused on the comparison of the composition of FVCs from the hydrosols and the lipophilic layer obtained by both extraction methods. The compositions of the LL of all 10 *Veronica* species studied that will be used for comparison were published in our previous work [6].

## 2. Results

### 2.1. Free Volatile Compounds of Ten Veronica Species

Free volatile compounds (FVCs) from the hydrosols of 10 Croatian *Veronica* species were isolated by classical hydrodistillation (HD) and green microwave-assisted extraction (MAE). The extracts were analyzed by GC-MS, and the results are presented in Table 1 and Table 2. In these tables, the identified compounds are divided into the following classes according to the time of their appearance on the nonpolar capillary column (VF5-ms): monoterpene hydrocarbons, oxygenated monoterpenes, sesquiterpene hydrocarbons, oxygenated sesquiterpenes, phenolic compounds, and a common group of acids, alcohols, and esters. 

#### 2.1.1. Free Volatile Compounds of the Hydrosols Obtained by Hydrodistillation

The composition of the hydrosols obtained by hydrodistillation of the *Veronica* species studied is given in Table 1. In the presentation of the results, the components that constitute more than 10% of the hydrosols are highlighted.

The compounds *β*-ionone, benzene acetaldehyde, (*E*)-*β*-damascenone, and *E*-caryophyllene were detected in all 10 studied HD hydrosols (Table 1). The highest percentage (13.78%) of identified *β*-ionone is present in *V. officinalis* L. while the highest relative abundance (25.65%) of (*E*)-*β*-damascenone was isolated from the hydrosols of *V. anagallis-aquatica* L. The content of benzene acetaldehyde is highest in *V. anagaloides* Guss. (26.52%) and *V. catenata* (26.31%) (Table 1). In addition, the compound α-muurolol is significantly present (14.88%) in the composition of *V. catenata*. Oxygenated sesquiterpenes are also strongly represented in *V. cymbalaria* with a percentage of 46.20%, while the most abundant compound in this species is the oxygenated sesquiterpene caryophyllene oxide with abundance 26.24%. Caryophyllene oxide is also significantly represented in *V. persica* with a percentage of 22.73% and in *V. acinifolia* L. with 26.43% (Table 1). Caryophyllene oxide was isolated in all HD hydrosols except in the hydrosol of *V. catenata* (Table 1). The compounds (*E*)-*β*-damascenone (10.46%), *E*-caryophyllene (15.43%), and caryophyllene oxide (12.15%) accounted for a significant proportion of the hydrosol composition of *V. polita* (Table 1).

Benzene acetaldehyde (13.52%), isopentyl acetate (10.41%), and (*E*)-*β*-damascenone (11.21%) are the most abundant compounds in *V. arvensis*, and all these compounds form a common class of acids, alcohols, and esters. This class accounts for 50.10% of all identified compounds in the composition of *V. arvensis* hydrosols (Table 1). The composition of *V. hederifolia L*. is dominated by benzaldehyde (16.32%) and the only monoterpene hydrocarbon identified is *α*-thujene (11.68%). The phenolic compound 2-methoxy-4-vinylphenol was isolated only in *V. officinalis* in both extraction methods, with the same percentage of 11.12% (Table 1 and Table 2).

#### 2.1.2. Free Volatile Compounds of the Hydrosols Obtained by Microwave-Assisted Extraction

The compounds *β*-ionone (17.78%), caryophyllene oxide (15.91%), and (*E*)-*β*-damascenone (11.34%) dominate in the composition of *V. officinalis* hydrosols obtained from MAE (Table 2). These three compounds are present in almost all isolates of *Veronica* species obtained via MAE. The exception is (E)*-β*-damascenone, which was not isolated from the hydrosols of *V. catenata* and *V. cymbalaria*. The peculiarity in the composition of hydrosols of these species is the high content of *α*-muurolol, which was 35.12% and 15.17% in *V. catenata* and *V. cymbalaria,* respectively (Table 2).

Linalool was detected in nine hydrosols obtained by MAE ranging from 0.83% (*V. anagalloides*) to 7.53% (*V. arvensis*). An exception was *V. acinifolia* in whose hydrosol linalool was not detected (Table 2). *E*-caryophyllene was also identified in nine MAE isolates except in hydrosol obtained from *V. arvensis*. The phenolic compound *p*-vinyl guaiacol was the most abundant (6.43%) in the hydrosol of *V. arvensis*. The monoterpenic hydrocarbon *β*-phellandrene was detected only in the hydrosol MAE of *V. arvensis*, with a percentage of 7.52% (Table 2). In addition, methyl eugenol was most frequently detected (18.35%) in *V. anagalloides*, while the *trans*-*p*-mentha-1(7),8-dien-2-ol was identified (3.22%) only in *V. anagallis-aquatica* (Table 2).

### 2.2. Comparison of the Composition of Free Volatile Compounds from Hydrosols and Lipophilic Layer

**Figure 1:**
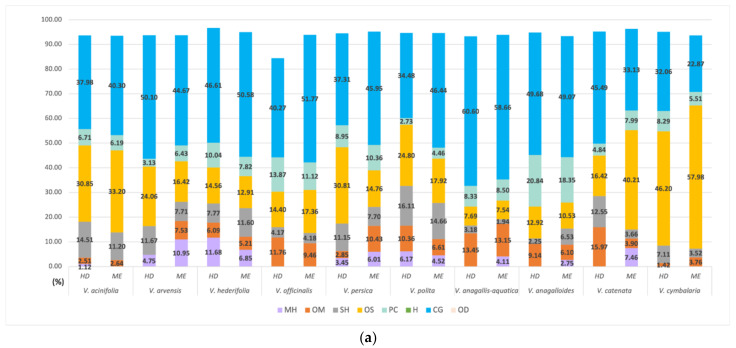

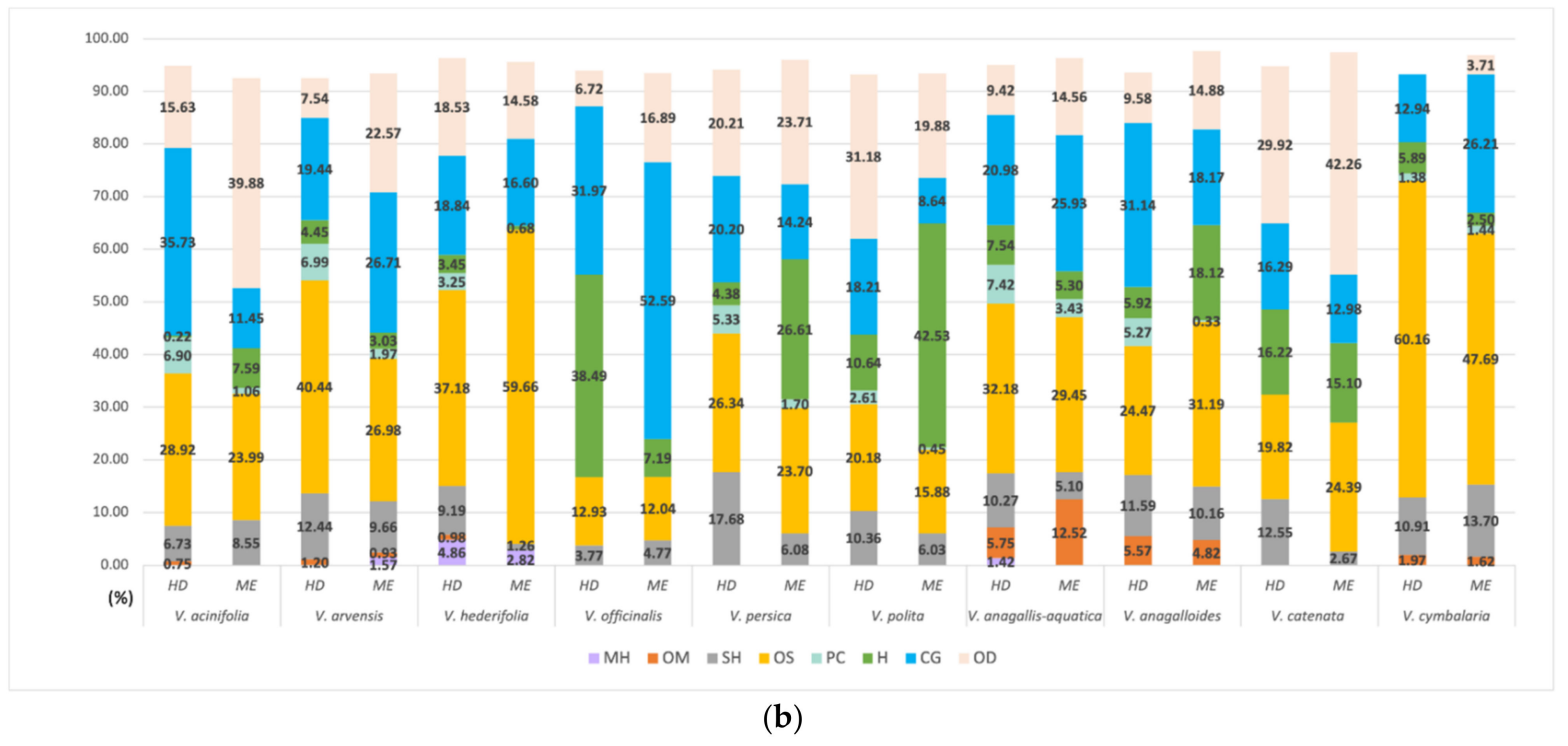
Relative content of hydrosol volatiles in 10 *Veronica* species divided into eight categories: monoterpene hydrocarbons (MH), oxygenated monoterpenes (OM), sesquiterpene hydrocarbons (SH), oxygenated sesquiterpenes (OS), oxygenated diterpene (OD), phenolic compounds (PD), hydrocarbons (H), and a common group (CG) of acids, alcohols, and esters. (**a**) categories for hydrosols from HD and MAE; (**b**) categories for LLs from HD and MAE.

**Figure 2:**
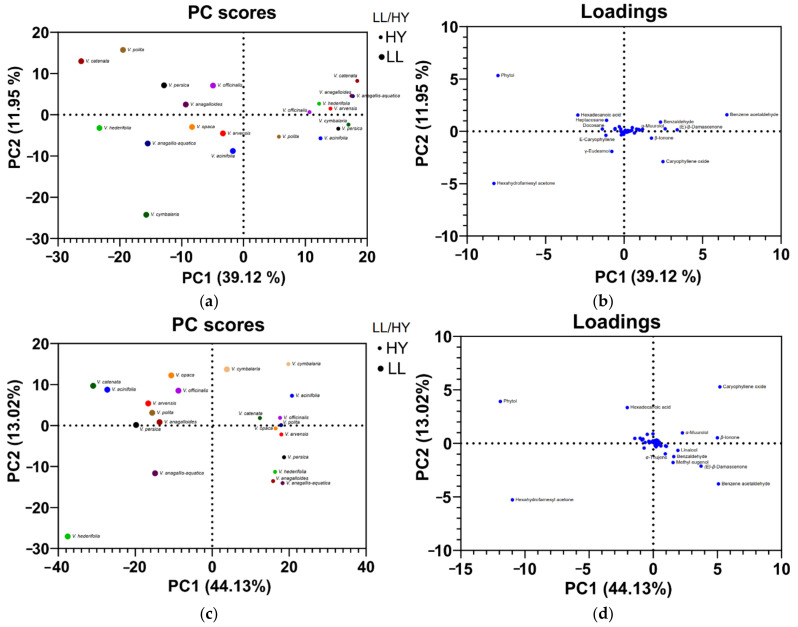
PCA analyses of volatile compounds of 10 *Veronica* species – hydrodistillation (HD) and microwave-assisted extraction (MAE). (**a**) PCA score plot for LLs and hydrosols (HY) from HD method; (**b**) PCA loading plots of volatiles from the first and second principal component for the LLs and hydrosols from HD method; (**c**) PCA score plot for LLs and hydrosols from MAE method; (**d**) PCA loading plots of volatiles from the first and second principal component for the LLs and hydrosols from the MAE method.

The data for the lipophilic layer from both extractions (essential oils) were taken from the previously published paper by Dunkić et al. [6] and were arranged in Figure 1 to provide a better preview of the differences (and similarities) between the FVCs from hydrosols (Figure 1a) and lipophilic layer (LL) (Figure 1b). The results show that hydrosols of most *Veronica* species studied contain more phenolic and oxygenated compounds (from the common group of acids, alcohols, and esters), and LL has a higher relative proportion of nonpolar compounds, such as oxygenated diterpenes and oxygenated sesquiterpenes (Figure 1a,b). Looking at the categories for hydrosols, the relative proportion of oxygenated compounds (belonging to the general group and to the phenol group) is similar in the majority of the samples studied by both methods. This could indicate that the quality of the extract is similar in both methods, but to investigate this probability for sure, the biological activity of the extracts should be studied and compared. This will be the next step in our research on this topic. PCA analyses were performed for volatile compounds from LL and hydrosols with an amount greater than 1%. Separate analyses were carried out for the classical (Figure 2a,b) and microwave extraction methods (Figure 2c,d). Data for FVCs composition from LL was taken from a previously published paper by Dunkić et al. [6]. PC1 and PC2 for volatile compounds from HD (Clevenger extraction) explained 51.07% of the variance. PC1 and PC2 for volatile compounds from MAE explained 56.07% of the variance. For both PCA analyses, it can be seen that there is a very clear difference between FVCs composition of LLs and hydrosols. On the PC score plot for Clevenger extraction, all hydrosol samples from investigated species are grouped on the right side of the score plot (positive region of PC1) and all LL samples are on the left side of the score plot (negative region of PC1) (Figure 2a,b). From the Loadings plot, it can be seen that hydrosols are characterized mostly by benzene acetaldehyde, benzaldehyde, *β*-ionone and caryophyllene oxide. LL samples are characterized mainly by phytol, hexadecanoic acid, hexahydrofarnesyl acetone, heptacosane and *γ*-eudesmol. The difference between the FVC composition of two investigated fractions can also be seen on the PC score plot for the MAE (Figure 2c,d). All hydrosol samples are on the right side of the score plot and all LL samples are on the left side of the score plot. Only one LL sample from the species *V. cymbalaria* is on the right side of the graph, but it is also closer to the LL cluster than the hydrosol cluster. From the loadings plot, it can be seen that hydrosols extracted by the MAE are characterized mostly by benzene acetaldehyde, benzaldehyde, *β*-ionone and caryophyllene oxide, the same compounds as in the hydrosols from HD extraction, with the additional compounds, methyl eugenol, (*E*)-*β*-damascenone and α-muurolol. The LL samples from the MAE were characterized mainly by phytol, hexadecanoic acid and hexahydrofarnesyl acetone, as were the LL samples from the HD extraction.

### 2.3. Antioxidant Activity

The antioxidant activity of the extracted volatile compounds of the hydrosols was analyzed using two methods, ORAC and DPPH, and the results are presented in the Table 3 and Table 4. Hydrosols from both extraction techniques, Clevenger and microwave-assisted hydrodistillation, were tested by the above-mentioned methods. *V. officinalis* had the highest activity in ORAC for both extraction methods (217.54 ± 14.98 µmol TE/mL for Clevenger extraction and 359.9 ± 44.40 µmol TE/mL for microwave extraction (Table 3). Some other species also showed higher antioxidant activity in addition to the one mentioned. These are *V. cymbalaria, V. anagallis-aquatica* and *V. anagalloides* for Clevenger extracted hydrosols, and *V. hederifolia* and *V. anagallis-aquatica* for the microwave-assisted extracted hydrosols (Table 3). Results for DPPH antioxidant activity method also showed that *V. officinalis* hydrosol has the highest activity, for both extraction methods (Table 4). Besides this species, higher antioxidant activity showed *V. acinifolia, V. arvensis* and *V. cymbalaria* for Clevenger extracted hydrosols (Table 4). In microwave-assisted extracted hydrosols, *V. cymbalaria* showed higher activity than other tested species, alongside *V. officinalis* (Table 4).

## 3. Discussion

Support for the use of herbal products in classical medicine has been confirmed by several clinical studies, so the use of herbal medicines is not a placebo medicine, but has a scientific basis [2]. Products with potential pharmaceutical effects are specialized plant metabolites. These metabolites, mostly consisting of monoterpenes and sesquiterpenes, are mainly lipophilic compounds that have an affinity for biomembranes, i.e., they bind to cell membranes. Depending on their concentration, these phytochemicals can alter membrane fluidity and increase permeability, allowing them to enter the cell and trigger various biological responses [2].

Some of the secondary or specialized metabolites of plants can be FVCs obtained by classical and/or green extraction. These extracts consist of volatile specialized metabolites isolated in the lipid part and components soluble in the water part of the extract-hydrosols. In this study, the composition of hydrosols of 10 *Veronica* from Croatia was presented. In addition, the compositions of hydrosols obtained by HD and MAE were compared.

According to Xue et al., the genus *Veronica* is a clinically important genus of the family Plantaginaceae [5]. Due to the richness of specialized metabolites, *Veronica* species can be considered to be a good candidate for pharmacological applications [4].

Common volatile compounds of all HD-hydrosols of investigated *Veronica* species are *β*-ionone, benzene acetaldehyde, (*E*)-*β*-damascenone, and *E*-caryophyllene (Table 1 and Table 2). These compounds have been shown to have beneficial effects on human health. In addition to the antibacterial and antifungal activities already known, each of these compounds has been shown to have a specific biological function [14,15]. For example, *β*-ionone has the ability to effectively suppress lung carcinogenesis triggered by benzo(a)pyrene [14]. The biosynthetic pathway of *β*-damascenone and *β*-ionone is specific, as *β*-damascenone is formed from xanthophyll, which gives rise to *β*-carotene, and *β*-ionone is formed by splitting *α*- and *β*-carotene [16].

Benzene acetaldehyde was also detected in the flower heads of the pharmaceutically important species *Arnica chamissonis* and in buckwheat (*Fagopyrum esculentum* Moench) seeds [17,18]. Since all our samples of *Veronica* species were collected during the flowering season, it is possible that most of this compound amount was isolated from the flowers. In addition to benzene acetaldehyde, another important aldehyde is benzaldehyde, which is detected mainly in the HD hydrolates of *V. hederifolia* (16.32%, Table 1) and *V. cymbalaria* (14.98%, Table 2). Benzaldehyde is the simplest and most commonly used aromatic compound with antifungal activity. Like many natural compounds, benzaldehyde is environmentally safe due to its biodegradability [19,20].

Another important compound is 2-methoxy-4-vinylphenol, which was isolated only in the hydrosols of *V. officinalis* in both extraction methods with the same percentage of 11.12% (Table 1 and Table 2). This phenolic constituent has a wide range of biological activities, and from the pharmacological point of view, its anti-inflammatory and analgesic activity is particularly important [21,22].

In the composition of hydrosols from MAE, caryophyllene oxide was detected in all isolates from MAE, in addition to the already mentioned *β*-ionone, which also dominates in the isolates from HD (Table 2). Preliminary pharmacological studies have shown that caryophyllene oxide administered intraperitoneally has analgesic and anti-inflammatory effects [23].

An important constituent of the MAE-hydrosols of the studied *Veronica* species is *α*-muurolol, which is the most abundant compound in the composition of *V. catenata*, with a content of 35.12% (Table 2). The essential oil of *Chamaecyparis formosensis* Matsum shows significant activity against mold, and one of the main bioactive components in the composition of the oil is α-muurolol [24]. Linalool, a commercially important constituent [25], has been identified in almost all MAE-hydrosols and is the only one not found in *V. acinifolia* species (Table 2).

According to Garagounis et al. (2021), it is not only important to determine the phytochemical composition of plant isolates, but also to further explore their biological activity for possible pharmaceutical use [1]. Our team continues to carry out biological research on hydrosols from the *Veronica* species to this end.

As for the differences between the two methods used in this study to obtain FVCs, there are similar comparisons in other plant species. Smadja et al. compared essential oils (EOs) from different aromatic herbs (*Ocimum basilicum* L., *Mentha crispa* L., and *Thymus vulgaris* L.) extracted with classical hydrodistillation and solvent-free microwave extraction [26]. They concluded that solvent-free microwave extraction is a good alternative for EO extraction because it yields more valuable EOs (EOs extracted with microwave extraction were richer in eugenol). Costa et al. investigated antimicrobial and antifungal activity of oregano (*Origanum glandulosum* Desf.) EO and compared the activities for two extraction methods, hydrodistillation and microwave extraction [27]. Their results showed that EOs extracted by solvent-free microwave extraction had better antimicrobial activity and this creates the possibility of using solvent-free microwave extraction as an alternative method for producing active essential oils. Hamedi et al. compared EO from rosemary (*Rosmarinus officinalis* L.) extracted by classical hydrodistillation and microwave-assisted extraction [28]. Their results also suggest that microwave extraction is a good alternative for hydrodistillation, because in their research, the relative percentage of oxygenated compounds was higher and the relative percentage of monoterpene hydrocarbons was lower when using the “green” extraction method. They concluded that higher quality EOs were extracted by the MAE method [28]. Khanavi et al. also investigated difference in composition of EOs extracted with classical and microwave extraction method for *Coriandrum sativum* L. EOs and the difference in antimicrobial activity between the extracts and showed some different results than previously reported [29]. EOs from HD had better antimicrobial activity, although the most important compound for this EOs (linalool) is similar in relative percentage for both methods. In our results for antioxidant activity for both methods of antioxidant activity determination, six species showed higher antioxidant activity with MAE extracts, so this may suggest that microwave-assisted extraction yields compounds with higher biological activities. This hypothesis needs to be investigated with other assays for biological activity such as antiproliferative, antimicrobial and antifungal.

PCA analysis is often used when detecting potential phytotaxonomic markers for specific genera or species, as numerous other experiments showed [30,31,32,33]. In this research, we did not use PCA for this purpose but to discriminate the composition of free volatile compounds in LLs and hydrosols. The difference in these two extracts is visible on the PCA Score plots. LL samples are characterized mainly by phytol, hexadecanoic acid, hexahydrofarnesyl acetone, heptacosane and γ-eudesmol, while hydrosols are characterized mostly by benzene acetaldehyde, benzaldehyde, *β*-ionone and caryophyllene oxide. These compounds could be helpful in phytotaxonomic studies of this genus. Current findings will be used in future research on chemophenetic (phytotaxonomic) markers among the volatiles of the genus *Veronica*.

The highest antioxidant activity in both methods and both extraction procedures was expected for the hydrosol of *V. officinalis*, since it is known that this species has been used in folk medicine for a long time and many studies support its biological activity [34,35,36]. Looking at the composition tables, this activity could be due to the relatively high content of phenolic compounds and the group of acids, esters and alcohols (Table 1). *Veronica cymbalaria* also showed high activity of hydrosols from both extraction methods. This could be due to the relatively high percentage of oxygenated sesquiterpenes in the composition of hydrosols (Figure 1a,b). Our results are in accordance with other reported findings. Harput et al. reported the highest total phenolic content for *V. officinalis* (200.20 mg/g). They also reported the strongest antioxidant activity for aqueous extracts of *V. officinalis* (IC50 54.19 µg/mL). Other high antioxidant activities of phenolic extracts of *Veronica* species have been reported. For example, research by Sharifi-Rad et al. showed DPPH antioxidant activity for methanol extract of aerial parts of *V. persica* to be IC50 30 µg/mL [37]. Dunkić et al. reported even higher antioxidant activity for methanol extracts of flowers of *V. spicata* with IC50 8.21 µg/mL [38]. Živković et al. investigated antioxidant activity of three *Veronica* species and reported the highest antioxidant activity for *V. teucrium* 70% aqueous acetone extracts (IC50 12.58 µg/mL) [39]. Comparing the results for the antioxidant activity of hydrosols and phenolic extracts of *Veronica* species, it is evident that phenolic extracts exert higher antioxidant activity, but this might be due to the relatively low concentrations of the active compounds in the hydrosols in general. Comparing the antioxidant activities of the *Veronica* species that showed the highest activity (*V. officinalis* and *V. cymbalaria*) with antioxidant activity of olive hydrosols, it can be concluded that they exert similar antioxidant activity to the olive hydrosols [40]. All these results show that speedwells should be further researched for their in vivo antioxidant activities or for potential usage in food preservations.

## 4. Materials and Methods

### 4.1. Isolation of Free Volatile Compounds

Ten Croatian *Veronica* species were used in this study and the voucher specimens were deposited in the herbarium of the Laboratory of Botany (HPMF-HR) of the Faculty of Science, University of Split, Croatia, under the designation CROVeS-No-2021 (Table 5). Plant material (leaves, flowers, stems) was collected at the flowering stage from March to July 2021 and air-dried under controlled conditions (room conditions 25 °C in dark place for 10 days). For the isolation of free volatile compounds (FVCs), 30 to 50 g of the dried material was used.

### 4.2. Preparation of the Samples and Analyses of Hydrosols by Gas Chromatography and Mass Spectrometry

Samples of 20 hydrosols (10 obtained by HD and 10 obtained by MAE) for analysis were prepared by adding 2 g of hydrosol to a glass bottle and capping it with a metal cap. The sample prepared in this way was placed in a water bath at 40 °C for 20 min so that the FVCs evaporate from the water. The process takes an additional 20 min to allow the FVCs to adsorb to the resin filament of the headspace needle that was injected through the septum of the bottle cap at the beginning of sample preparation.

Injection of hydrosols was carried out with a headspace injection needle and there was no split ratio (splitless mode). The injection needle of FVCs thus collected from the hydrososol was then inserted into a GC inlet and left there for 20 min to ensure that all FVCs from the resin filament were resorbed into the injection liner.

Gas chromatographic analyses were performed using a gas chromatograph (model 3900; Varian Inc., Lake Forest, CA, USA) equipped with a flame ionization detector and a mass spectrometer (model 2100T; Varian Inc., Lake Forest, CA, USA), nonpolar capillary column VF-5ms (30 m × 0.25 mm i.d., coating thickness 0.25 μm, Palo Alto, CA, USA), and polar CP Wax 52 (30 m × 0.25 mm i.d., coating thickness 0.25 μm, Palo Alto, CA, USA) according to the method described in [34,35,36]. The chromatographic conditions for the analysis of the hydrosol fraction were the same as described in the article by Dunkić et al. [6,13,37]. In that study, the oil fractions of the same distillates were analyzed [6].

The individual peaks for all samples were identified by a comparison of their retention indices of n-alkanes to those of authentic samples and the studies [13,41], a comparison to our libraries from previous work, and a comparison to other previously published material for *Veronica* species [35,42,43]. The results are expressed as the mean value of three analyses with the standard deviation.

### 4.3. Antioxidant Activity of Essential Oils and Hydrosols

#### 4.3.1. ORAC (Oxygen Radical Absorbance Capacity)

The assay was performed in a Tecan Infinite 200 PRO spectrophotometer (Tecan Trading AG, Männedorf, Switzerland), using 96-well black polystyrene microtiter plates (Porvair Sciences, Leatherhead, UK) according to a method described by Nazlic et al. [44]. Each reaction contained 180 µL of fluorescein (1 µM), 70 µL 2,2′-Azobis(2-methyl-propionamidine) dihydrochloride (AAPH, Acros Organics) (300 mM), and 30 µL of plant extract or reference standard Trolox (6.25–50 µM) (Sigma-Aldrich, St. Louis, MO, USA). All experimental solutions were prepared in a phosphate buffer (0.075 mM, pH 7.0). We used absolute hydrosol and dissolved it in a phosphate buffer, so for experiments we had absolute hydrosol and 50% (2× diluted) hydrosol for every species and every extraction method. The measurements were performed in triplicate by a method described in Fredotovic et al. [45]. The ORAC values of hydrosols are expressed as µmol of Trolox equivalents (TE) per mL of the tested hydrosol sample for the concentration of 10 mg/mL. The results were obtained from three independent experiments.

#### 4.3.2. Measurement of the DPPH Radical Scavenging Activity

The antioxidant capacity of the extracts was assessed using the DPPH method previously described by Mensor et al. and Payet et al. [46,47]. This method is based on the reduction of alcoholic DPPH (2,2-diphenyl-1-picrylhydrazyl) solution (Sigma-Aldrich) in the presence of a hydrogen-donating antioxidant using 96-well microtiter plates. Absolute (undiluted) hydrosols were used for the reaction. We pipetted 100 µL methanol (Kemika, Zagreb, Croatia) and 200 µL standard and/or sample into each well. We prepared serial dilutions of standard and samples by pipetting 100 µL from the first row with a multichannel pipette into the wells in the second row and so on to the last row, where 100 µL of the solution was ejected after mixing. In the first column, in 96-well plates, a blank sample was always added (distilled water) and in the second column, Trolox standard of a 200 µM concentration. The reaction started by adding 100 µL of a methanolic solution of DPPH (200 µM) to each well. The initial absorbance at 517 nm was measured immediately (it should be around 1.1). After 30 min incubation, the absorbance was measured again, and the percentage of DPPH inhibition was calculated according to the following formula by Yen and Duh [48]:

% inhibition = ((AC(0) − AA(t))/AC(0)) × 100,

where AC(0) is the absorbance of the control at t = 0 min, and AA(t) is the absorbance of the antioxidant at t = 30 min. All measurements were performed in triplicate.

Because of the data from other relevant literature, we expressed results as IC50 values in mg of volatile compounds/mL of hydrosol.

### 4.4. PCA Analyses

Statistical analysis was performed in GraphPad Prism Version 9 (GraphPad Software, San Diego, CA, USA). All data in the tables are expressed as the mean ± SD (n = 3). Data included in the PCA analyses were obtained from the GC-MS analyses. PCA analyses were performed for VCs with amounts greater than 1%.

## 5. Conclusions

The composition of free volatile compounds (FVC) from hydrosols of 10 *Veronica* species was analyzed using gas chromatography and mass spectrometry. Two extraction methods were used, classical hydrodistillation and microwave-assisted extraction, and the composition of the compounds was compared. These volatile compounds belong to the specialized metabolites, and it is important to know their composition for further research on the pharmaceutical activity of *Veronica* species. Compared to the lipophilic phase, hydrosols are safer for any kind of human use due to their milder composition. In the part of the research focused on comparing the composition of FVCs from the hydrosols and the lipophilic layer obtained by both extraction methods, the results show that hydrosols contain more phenolic and oxygenated compounds. In the lipophilic layer, the relative percentage of nonpolar compounds, such as oxygenated diterpenes and oxygenated sesquiterpenes, is higher. Considering that both extraction methods isolated the same major constituents, our results also suggest that microwave extraction is a good alternative for the extraction of FVCs. This conclusion is also supported with the results for antioxidant activity, according to which hydrosols for six out of ten investigated species resulted in better activity for MAE extracts for both antioxidant methods. Comparing the tested antioxidant activity of all hydrosols, it can be concluded that besides *V. officinalis*, some other *Veronica* species also showed antioxidant activity. These are *V. cymbalaria*, *V. hederifolia*, *V. anagallis-aquatica* and *V. anagalloides*. Further research should be based on the study of other biological activities of these extracts for possible pharmaceutical use.

## Figures and Tables

**Table 1:** Free volatile compounds (FVCs) of the hydrosols obtained by hydrodistillation from the aerial parts of *Veronica* species.

			*V. acinifolia*	*V. anagallis-aquatica*	*V. anagalloides*	*V. catenata*	*V. arvensis*	*V. cymbalaria*	*V. hederifolia*	*V. officinalis*	*V. persica*	*V. polita*
**Component**	**RI^a^**	**RI^b^**	**FVC ± SD**	**FVC ± SD**	**FVC ± SD**	**FVC ± SD**	**FVC ± SD**	**FVC ± SD**	**FVC ± SD**	**FVC ± SD**	**FVC ± SD**	**FVC ± SD**
**Monoterpene hydrocarbons**			**1.12**	-	-	-	**4.75**	-	**11.68**	-	**3.45**	**6.17**
*α*-Thujene	924	1012	1.12 ± 0.02	-	-	-	4.75 ± 0.01	-	11.68 ± 0.01	-	3.45 ± 0.01	6.17 ± 0.01
**Oxygenated monoterpenes**			**2.51**	**13.45**	**9.14**	**15.97**	**-**	**1.42**	**6.09**	**11.76**	**2.85**	**10.36**
γ-Terpinene	1057	1225	-	-	3.11 ± 0.01	-	-	-	-	-	-	-
Linalool	1095	1506	0.95 ± 0.01	3.35 ± 0.01	0.76 ± 0.01	4.76 ± 0.01	-	1.15 ± 0.01	3.54 ± 0.01	4.21 ± 0.01	1.94 ± 0.01	2.35 ± 0.01
*β*-Thujone	1121	1438	-	-	-	-	-	-	-	7.55 ± 0.01	-	-
Terpinen-4-ol	1174	1686	0.75 ± 0.01	4.78 ± 0.01	4.54 ± 0.1	3.22 ± 0.01	-	0.27 ± 0.01	-	-	0.91 ± 0.01	8.01 ± 0.01
*α*-Terpineol	1184	1660	0.81 ± 0.1	-	-	7.54 ± 0.01	-	-	2.55 ± 0.03	-	-	-
*trans-p*-Mentha-1(7),8-dien-2-ol	1187	1803	-	5.32 ± 0.01	0.73 ± 0.15	0.45 ± 0.03	-	-	-	-	-	-
**Sesquiterpene hydrocarbons**			**14.51**	**3.18**	**2.25**	**12.55**	**11.67**	**7.11**	**7.77**	**4.17**	**11.15**	16.11
*α*-Copaene	1377	1484	0.75 ± 0.1	-	-	-	-	-	-	-	-	-
*β*-Elemene	1389	1593	-	-	-	-	-	-	-	-	-	0.68 ± 0.03
*E*-Caryophyllene *	1424	1585	7.15 ± 0.01	0.78 ± 0.01	1.31 ± 0.01	4.11 ± 0.01	1.63 ± 0.01	1.05 ± 0.02	4.11 ± 0.01	1.85 ± 0.01	1.44 ± 0.01	15.43 ± 0.01
*allo*-Aromadendrene	1465	1662	-	-	0.56 ± 0.01	1.23 ± 0.01	2.52 ± 0.01	-	2.26 ± 0.01	2.32 ± 0.01	6.52 ± 0.01	-
*β*-Chamigrene	1478	1724	-	-	-	-	1.62 ± 0.1	-	-	-	-	-
Germacrene D	1481	1692	6.86 ± 0.01	0.95 ± 0.01	0.38 ± 0.02	3.87 ± 0.02	3.47 ± 0.1	3.72 ± 0.01	0.75 ± 0.02	-	0.63 ± 0.01	-
*δ*-Selinene	1492	1756	2.75 ± 0.01	1.45 ± 0.01	-	3.34 ± 0.01	2.43 ± 0.01	0.78 ± 0.01	0.65 ± 0.1	-	1.55 ± 0.01	-
*δ*-Cadinene	1517	1745	-	-	-	-	-	1.56 ± 0.01	-	-	1.01 ± 0.01	-
**Oxygenated sesquiterpenes**			**30.85**	**7.69**	**12.92**	**16.42**	**24.06**	**46.2**	**14.56**	**14.4**	**30.81**	**24.8**
Spathulenol	1577	2101	1.15 ± 0.01	0.44 ± 0.01	1.32 ± 0.01	-	-	-	0.88 ± 0.01	-	-	-
Caryophyllene oxide *	1581	1955	26.43 ± 0.01	3.56 ± 0.01	6.35 ± 0.01	-	7.92 ± 0.01	26.24 ± 0.01	6.32 ± 0.01	8.15 ± 0.01	22.73 ± 0.01	12.15 ± 0.01
Viridiflorol	1592	2099	-	0.75 ± 0.01	-	-	-	-	-	-	-	0.82 ± 0.03
γ-Eudesmol	1632	2175	-	-	-	-	6.38 ± 0.01	0.48 ± 0.01	-	0.56 ± 0.01	-	1.31 ± 0.01
α-Muurolol	1645	2181	-	0.62 ± 0.01	3.22 ± 0.01	14.88 ± 0.01	9.76 ± 0.01	15.17 ± 0.01	2.48 ± 0.01	1.02 ± 0.01	7.21 ± 0.01	0.75 ± 0.01
α-Cadinol	1655	2208	-	0.62 ± 0.01	3.22 ± 0.01	14.88 ± 0.01	-	15.17 ± 0.01	-	0.98 ± 0.1	0.34 ± 0.05	-
α-Bisabolol	1685	2210	0.74 ± 0.01	-	0.58 ± 0.1	-	-	3.36 ± 0.01	3.13 ± 0.01	1.03 ± 0.01	-	0.63 ± 0.01
α-Bisabolol oxide	1748	2511	0.88 ± 0.07	-	-	-	-	-	-	0.81 ± 0.01	-	0.88 ± 0.01
Hexahydrofarnesyl acetone *	1839	2113	1.65 ± 0.01	2.32 ± 0.01	2.45 ± 0.01	1.54 ± 0.01	-	0.95 ± 0.01	1.75 ± 0.01	1.85 ± 0.01	0.53 ± 0.01	8.26 ± 0.01
**Phenolic compounds**			**6.71**	**8.33**	**20.84**	**4.84**	**3.13**	**8.29**	**10.04**	**13.87**	**8.95**	**2.73**
Thymol *	1289	2154	-	-	-	-	-	3.44 ± 0.01	4.12 ± 0.02	-	-	-
*p*-Vinyl guaicol	1313	2156	1.43 ± 0.01	3.91 ± 0.01	1.52 ± 0.01	4.10 ± 0.01	2.11 ± 0.01	-	3.68 ± 0.01	2.75 ± 0.01	6.04 ± 0.01	2.73 ± 0.01
2-Methoxy-4-vinylphenol	1317	2145	-	-	-	-	-	-	-	11.12 ± 0.01	-	-
Thymol acetate	1349	-	0.85 ± 0.01	-	-	-	-	-	-	-	-	-
Methyl eugenol	1403	2005	4.43 ± 0.01	4.42 ± 0.01	16.71 ± 0.01	0.74 ± 0.01	1.02 ± 0.05	-	1.38 ± 0.01		2.20 ± 0.01	-
(*Z*)-Methyl isoeugenol	1451	2070	-	-	2.61 ± 0.01	-	-	4.85 ± 0.01	0.86 ± 0.05	-	0.71 ± 0.01	-
**Acids, alcohols and esters**			**37.98**	**60.6**	**49.68**	**45.49**	**50.1**	**32.06**	**46.61**	**40.27**	**37.31**	**34.48**
Isopentyl acetate	863	1127	-	-	-	-	10.41 ± 0.01	-	-	-	-	-
Benzaldehyde	952	1508	5.89 ± 0.1	9.32 ± 0.01	4.17 ± 0.01	9.47 ± 0.01	-	14.98 ± 0.01	16.32 ± 0.01	2.15 ± 0.01	-	-
Benzene acetaldehyde	1036	1633	7.38 ± 0.01	16.78 ± 0.01	26.52 ± 0.01	26.31 ± 0.01	13.52 ± 0.01	12.84 ± 0.01	9.31 ± 0.01	10.69 ± 0.01	11.03 ± 0.01	8.41 ± 0.01
*n*-Nonanal	1100	1389	1.73 ± 0.02	-	2.38 ± 0.01	-	2.56 ± 0.01	-	4.37 ± 0.01	0.46 ± 0.1	1.37 ± 0.01	0.72 ± 0.01
Hexyl 2-methyl butanoate	1233	1425	1.43 ± 0.1	-	3.97 ± 0.01	-	-	-	-	-	0.46 ± 0.1	-
n-Decanol	1266	1711	0.64 ± 0.01	-	1.46 ± 0.01	-	1.54 ± 0.01	-	-	-	2.22 ± 0.01	-
*2,4*-Decadienal	1304	1764	-	-	-	-	-	-	-	-	0.74 ± 0.02	-
(*E*)-*β*-Damascenone	1384	1819	7.86 ± 0.01	25.65 ± 0.01	3.12 ± 0.01	2.15 ± 0.01	11.21 ± 0.01	2.42 ± 0.01	7.42 ± 0.01	6.34 ± 0.01	9.32 ± 0.01	10.46 ± 0.01
*β*-Ionone	1487	1935	12.33 ± 0.1	8.85 ± 0.01	7.86 ± 0.01	7.56 ± 0.01	8.94 ± 0.01	1.82 ± 0.01	8.53 ± 0.01	13.78 ± 0.01	11.73 ± 0.01	11.25 ± 0.01
Methyl salicylate	1188	1755	-	-	-	-	-	-	-	4.93 ± 0.01	-	-
Hexadecanoic acid *	1959	2912	0.72 ± 0.05	-	0.20 ± 0.05	-	1.92 ± 0.01	-	0.66 ± 0.01	1.92 ± 0.01	0.44 ± 0.01	3.64 ± 0.01
**Total identification (%)**			**93.68**	**93.25**	**94.83**	**95.27**	**93.71**	**95.08**	**96.75**	**84.01**	**94.52**	**94.65**

Retention indices (RIs) were determined relative to a series of n-alkanes (C8–C40) on capillary columns VF5-ms (RI^a^) and CPWax 52 (RI^b^); identification method: RI, comparison of RIs with those in a self-generated library reported in the literature [13] and/or with authentic samples; comparison of mass spectra with those in the NIST02 and Wiley 9 mass spectral libraries; * injection with reference compounds; -, not identified; SD, standard deviation of triplicate analysis.

**Table 2:** Free volatile compounds (FVCs) of the hydrosols obtained by microwave extraction from the aerial parts of *Veronica* species.

			*V. acinifolia*	*V. anagallis-aquatica*	*V. anagalloides*	*V. catenata*	*V. arvensis*	*V. cymbalaria*	*V. hederifolia*	*V. officinalis*	*V. persica*	*V. polita*
**Component**	**RI^a^**	**RI^b^**	**FVC ± SD**	**FVC ± SD**	**FVC ± SD**	**FVC ± SD**	**FVC ± SD**	**FVC ± SD**	**FVC ± SD**	**FVC ± SD**	**FVC ± SD**	**FVC ± SD**
**Monoterpene hydrocarbons**			**-**	**4.11**	**2.75**	**7.46**	**10.95**	**-**	**6.85**	**-**	**6.01**	**4.52**
*α*-Thujene	924	1012	-	1.69 ± 0.01	-	7.46 ± 0.01	3.43 ± 0.01	-	6.85 ± 0.01	-	6.01 ± 0.01	4.52 ± 0.01
*α*-Pinene *	935	1017	-	2.42 ± 0.01	2.75 ± 0.01	-	-	-	-	-	-	-
*β* -Phellandrene	1002	1195	-	-	-	-	7.52 ± 0.01	-	-	-	-	-
**Oxygenated monoterpenes**			**2.64**	**13.15**	**6.1**	**3.9**	**7.53**	**3.76**	**5.21**	**9.46**	**10.43**	**6.61**
γ-Terpinene	1057	1225	-	2.65 ± 0.01	3.52 ± 0.01	-	-	-	-	-	-	-
Linalool	1095	1506	-	3.11 ± 0.01	0.83 ± 0.05	3.15 ± 0.01	7.53 ± 0.01	1.67 ± 0.03	5.21 ± 0.01	6.11 ± 0.01	6.68 ± 0.01	3.18 ± 0.01
*β*-Thujone	1121	1438	-	-	-	-	-	-	-	3.35 ± 0.01	-	-
Terpinen-4-ol	1174	1686	2.64 ± 0.01	4.17 ± 0.01	1.75 ± 0.01	0.75 ± 0.01	-	2.09 ± 0.01	-	-	3.75 ± 0.01	3.43 ± 0.01
*trans-p*-Mentha-1(7),8-dien-2-ol	1187	1803	-	3.22 ± 0.01	-	-	-	-	-	-	-	-
**Sesquiterpene hydrocarbons**			**11.2**	**1.94**	**6.53**	**3.66**	**7.71**	**3.52**	**11.6**	**4.18**	**7.7**	**14.66**
*α*-Copaene	1377	1484	-	-	-	-	0.63 ± 0.1	-	-	-	-	-
*β*-Elemene	1389	1593	4.84 ± 0.01	-	-	-	3.75 ± 0.01	-	1.76 ± 0.01	0.38 ± 0.01	0.98 ± 0.01	-
*E*-Caryophyllene *	1424	1585	3.34 ± 0.01	1.46 ± 0.01	1.24 ± 0.01	2.53 ± 0.01	-	1.32 ± 0.01	4.58 ± 0.01	3.37 ± 0.01	3.72 ± 0.01	11.74 ± 0.01
*allo*-Aromadendrene	1465	1662	0.67 ± 0.1	-	1.05 ± 0.03	1.13 ± 0.01	-	0.53 ± 0.1	0.86 ± 0.01	-	0.86 ± 0.01	0.72 ± 0.02
Germacrene D	1481	1692	2.35 ± 0.01	0.48 ± 0.1	3.23 ± 0.01	-	2.45 ± 0.01	1.67 ± 0.01	2.27 ± 0.01	-	0.75 ± 0.01	1.02 ± 0.01
*δ*-Selinene	1492	1756	-	-	1.01 ± 0.01	-	0.88 ± 0.01	-	2.13 ± 0.01	-	1.39 ± 0.01	1.18 ± 0.01
*δ*-Cadinene	1517	1745	-	-	-	-	-	-	-	0.43 ± 0.01	-	-
**Oxygenated sesquiterpenes**			**33.2**	**7.54**	**10.53**	**40.21**	**16.42**	**57.98**	**12.91**	**17.36**	**14.76**	**17.92**
Spathulenol	1577	2101	1.43 ± 0.01	1.04 ± 0.03	-	0.85 ± 0.15	-	-	-	-	-	-
Caryophyllene oxide *	1581	1955	28.22 ± 0.01	2.76 ± 0.01	6.43 ± 0.01	2.72 ± 0.03	13.66 ± 0.01	37.12 ± 0.01	5.51 ± 0.01	15.91 ± 0.01	10.17 ± 0.01	14.17 ± 0.01
Viridiflorol	1592	2099	-	-	-	-	-	4.67 ± 0.01	-	-	-	0.84 ± 0.03
γ-Eudesmol	1632	2175	-	-	-	-	0.74 ± 0.01	-	-	-	2.76 ± 0.01	-
α-Muurolol	1645	2181	-	3.28 ± 0.01	-	35.12 ± 0.01	2.02 ± 0.03	15.17 ± 0.01	1.91 ± 0.01	-	-	1.31 ± 0.01
α-Cadinol	1655	2208	-	-	-	-	-	-	0.83 ± 0.01	-	-	-
α-Bisabolol	1685	2210	2.67 ± 0.01	0.46 ± 0.01	1.72 ± 0.1	-	-	-	1.15 ± 0.01	0.17 ± 0.01	0.63 ± 0.01	0.43 ± 0.1
α-Bisabolol oxide	1748	2511	-	-	0.92 ± 0.05	-	-	-	0.67 ± 0.01	0.62 ± 0.05	0.54 ± 0.01	0.53 ± 0.01
Hexahydrofarnesyl acetone *	1839	2113	0.88 ± 0.01	-	1.46 ± 0.01	1.52 ± 0.01	-	1.02 ± 0.05	2.84 ± 0.01	0.66 ± 0.01	0.66 ± 0.1	0.64 ± 0.01
**Phenolic compounds**			**6.19**	**8.5**	**18.35**	**7.99**	**6.43**	**5.51**	**7.82**	**11.12**	**10.36**	**4.46**
Thymol *	1289	2154	-	-	-	-	-	3.83 ± 0.01	5.17 ± 0.01	-	-	-
*p*-Vinyl guaicol	1313	2156	-	1.85 ± 0.01	-	4.67 ± 0.01	6.43 ± 0.01	0.77 ± 0.03	2.65 ± 0.01	-	-	4.46 ± 0.01
2-Methoxy-4-vinylphenol	1317	2145	-	-	-	-	-	-	-	11.12 ± 0.01	-	-
Methyl eugenol	1403	2005	5.43 ± 0.01	6.65 ± 0.01	18.35 ± 0.01	3.32 ± 0.01	-	-	-	-	8.65 ± 0.01	-
(*Z*)-Methyl isoeugenol	1451	2070	0.66 ± 0.01	-	-	-	-	0.91 ± 0.01	-	-	1.71 ± 0.01	-
**Acids, alcohols and esters**			**40.3**	**58.66**	**49.07**	**33.13**	**44.67**	**22.87**	**50.58**	**51.77**	**45.95**	**46.44**
Isopentyl acetate	863	1127	-	-	-	-	3.78 ± 0.01	4.15 ± 0.01	-	-	-	-
Benzaldehyde	952	1508	-	5.42 ± 0.01	17.35 ± 0.01	1.77 ± 0.01	4.88 ± 0.01	3.28 ± 0.01	-	1.15 ± 0.01	4.09 ± 0.01	-
Benzene acetaldehyde	1036	1633	4.54 ± 0.01	18.34 ± 0.01	22.52 ± 0.01	5.56 ± 0.01	10.32 ± 0.01	5.47 ± 0.01	14.36 ± 0.01	9.69 ± 0.01	20.05 ± 0.01	10.43 ± 0.01
*n*-Nonanal	1100	1389	0.43 ± 0.01	-	-	-	3.13 ± 0.01	-	1.44 ± 0.01	0.77 ± 0.01	0.72 ± 0.1	8.11 ± 0.01
n-Decanol	1266	1711	3.76 ± 0.01	4.67 ± 0.01	-	2.52 ± 0.01	-	-	-	-	2.55 ± 0.01	-
(*E*)-*β*-Damascenone	1384	1819	10.04 ± 0.01	20.45 ± 0.1	1.52 ± 0.02	-	8.85 ± 0.01	-	23.86 ± 0.01	11.34 ± 0.01	2.05 ± 0.01	8.01 ± 0.01
*β*-Ionone	1487	1935	21.53 ± 0.01	9.78 ± 0.01	6.47 ± 0.01	10.43 ± 0.01	13.71 ± 0.01	9.11 ± 0.01	10.14 ± 0.01	17.78 ± 0.01	16.49 ± 0.01	19.21 ± 0.01
Methyl salicylate	1188	1755	-	-	-	-	-	-	-	3.93 ± 0.01	-	-
Hexadecanoic acid *	1959	2912	-	-	1.21 ± 0.01	12.85 ± 0.01	-	0.86 ± 0.1	0.78 ± 0.01	7.11 ± 0.01	-	0.68 ± 0.01
**Total identification (%)**			**93.53**	**93.9**	**93.33**	**96.35**	**93.71**	**93.64**	**94.92**	**93.89**	**95.21**	**94.61**

Retention indices (RIs) were determined relative to a series of n-alkanes (C8–C40) on capillary columns VF5-ms (RI^a^) and CPWax 52 (RI^b^); identification method: RI, comparison of RIs with those in a self-generated library reported in the literature [13] and/or with authentic samples; comparison of mass spectra with those in the NIST02 and Wiley 9 mass spectral libraries; * injection of reference compounds; -, not identified; SD, standard deviation of triplicate analysis.

**Table 3:** Antioxidant potential determined using the ORAC method of the hydrosols obtained by Clevenger and microwave-assisted extraction from the aerial parts of 10 *Veronica* species.

Species	Clevenger	Microwave
*V. acinifolia*	12.35 ± 2.14	3.98 ± 0.99
*V. anagallis-aquatica*	**70.72 ± 2.84**	**57.29 ± 1.17**
*V. anagalloides*	**77.88 ± 9.59**	37.17 ± 3.33
*V. catenata*	11.23 ± 1.41	30.03 ± 0.79
*V. arvensis*	28.35 ± 0.3	31.9 ± 1.02
*V. cymbalaria*	**102.28 ± 3.04**	27.06 ± 2.99
*V. hederifolia*	37.96 ± 3.31	**86.88 ± 5.14**
*V. officinalis*	**217.54 ± 14.98**	**359.9 ± 44.40**
*V. persica*	27.62 ± 0.21	38.23 ± 1.45
*V. polita*	17.005 ± 1.19	34.42 ± 1.77

ORAC, oxygen radical absorbance capacity, results expressed as µmol of Trolox equivalents (TE) per L of the tested hydrosol sample for the concentration of 10 mg/mL. Results with the highest activity are bolded.

**Table 4:** Antioxidant potential determined by DPPH method of the hydrosols obtained by Clevenger and microwave-assisted extraction from the aerial parts of 10 *Veronica* species.

Species	Clevenger	Microwave
*V. acinifolia*	**8.09 ± 0.94**	36.04 ± 11.19
*V. anagallis-aquatica*	57.02 ± 4.93	119.6 ± 10.7
*V. anagalloides*	43.42 ± 5.49	21.18 ± 8.95
*V. catenata*	75.27 ± 9.5	17.83 ± 7.44
*V. arvensis*	**7.83 ± 2.16**	15.03 ± 5.34
*V. cymbalaria*	**6.55 ± 0.98**	**7.92 ± 1.52**
*V. hederifolia*	40.39 ± 6.13	24.42 ± 3.14
*V. officinalis*	**4.43 ± 2.44**	**3.70 ± 1.35**
*V. persica*	17.34 ± 3.81	24.87 ± 4.20
*V. polita*	48.99 ± 8.39	89.11 ± 9.79

DPPH, results are expressed in IC50 value in mg of volatile compound/mL of hydrosol. Results with the highest activity are bolded.

**Table 5:** Details of the data collection and origin of the investigated 10 *Veronica* species.

Species	Locality	Latitude	Longitude	Altitude a.s.l. (m)	Voucher No.
*V. acinifolia* L.	Donji Karin	44°07′18.1″ N	15°36′13.7″ E	119	CROVeS-11-2021
*V. anagallis-aquatica* L.	Split	43°31′43.5″ N	16°28′45.2″ E	22	CROVeS-06-2021
*V. anagalloides* Guss.	Čikola River	43°49′36.2″ N	16°01′19.4″ E	45	CROVeS-07-2021
*V. catenata* Pennell	Trakošćan	46°15′30.3″ N	15°56′25.2″ E	240	CROVeS-09-2021
*V. arvensis* L.	Hvar Island	43°10′42.3″ N	16°36′43.6″ E	38	CROVeS-12-2021
*V. cymbalaria* Bodard	Murter Island	43°48′36.6″ N	15°35′07.4″ E	37	CROVeS-03-2021
*V. hederifolia* L.	Zagreb	45°49′40.4″ N	15°58′59.6″ E	192	CROVeS-14-2021
*V. officinalis* L.	Kamešnica Mt	43°42′38.7″ N	16°50′47.9″ E	1225	CROVeS-16-2021
*V. persica* Poir.	Samoborsko gorje	45°49′41.6″ N	15°40′32.9″ E	301	CROVeS-18-2021
*V. polita* Fr.	Kaštel Žegarski	44°09′26.1″ N	15°51′56.0″ E	53	CROVeS-19-2021

The FVCs were isolated using hydrodistillation in a Clevenger-type apparatus (Šurlan, Medulin, Croatia) and microwave-assisted extraction (Milestone ‘ETHOS X’ microwave laboratory oven, 1900 W maximum) for 2.5 h, using 30–50 g of dried plant material. The locations where the plant material was collected are presented in the Table 5. The working conditions for both isolation techniques are described in the study of Dunkić et al. [6]. The distillate consists of two layers: a lipophilic layer collected in a side tube using a pentane/diethyl ether trap, and a water layer (hydrosol). The composition of 20 hydrosols obtained, 10 of which were isolated by classical distillation and 10 by green extraction (microwave extraction), was further investigated.

## Data Availability

The samples and any additional research data are available from the authors on request.

## References

[1] Garagounis C., Delkis N., Papadopoulou K.K. (2021). Unraveling the Roles of Plant Specialized Metabolites: Using Synthetic Biology to Design Molecular Biosensors. New Phytol..

[2] Wink M. (2015). Modes of Action of Herbal Medicines and Plant Secondary Metabolites. Medicines.

[3] Tutin T.G., Heywood V.H., Burges N.A., Moore D.M., Valentine D.H., Walters S.M., Webb D.A. (1972). Flora Europaea.

[4] Salehi B., Shetty M.S., Anil Kumar N.V., Živković J., Calina D., Docea A.O., Emamzadeh-Yazdi S., Kılıç C.S., Goloshvili T., Nicola S. (2019). *Veronica* Plants—Drifting from Farm to Traditional Healing, Food Application, and Phytopharmacology. Molecules.

[5] Xue H., Chen K.X., Zhang L.Q., Li Y.M. (2019). Review of the Ethnopharmacology, Phytochemistry, and Pharmacology of the Genus *Veronica*. Am. J. Chin. Med..

[6] Dunkić V., Nazlić M., Ruščić M., Vuko E., Akrap K., Topić S., Milović M., Vuletić N., Puizina J., Jurišić Grubešić R. (2022). Hydrodistillation and Microwave Extraction of Volatile Compounds: Comparing Data for Twenty-One *Veronica* Species from Different Habitats. Plants.

[7] Rao B.R.R. (2013). Hydrosols and Water-Soluble Essential Oils: Medicinal and Biological Properties. Recent Progress in Medicinal Plants: Essential Oils I.

[8] D’Amato S., Serio A., López C.C., Paparella A. (2018). Hydrosols: Biological Activity and Potential as Antimicrobials for Food Applications. Food Control.

[9] Hamedi A., Moheimani S.M., Sakhteman A., Etemadfard H., Moein M. (2017). An Overview on Indications and Chemical Composition of Aromatic Waters (Hydrosols) as Functional Beverages in Persian Nutrition Culture and Folk Medicine for Hyperlipidemia and Cardiovascular Conditions. J. Evid.-Based Complement. Altern. Med..

[10] Ilieva Y., Dimitrova L., Georgieva A., Vilhelmova-Ilieva N., Zaharieva M.M., Kokanova-Nedialkova Z., Dobreva A., Nedialkov P., Kussovski V., Kroumov A.D. (2022). In Vitro Study of the Biological Potential of Wastewater Obtained after the Distillation of Four Bulgarian Oil-Bearing Roses. Plants.

[11] Shen X., Chen W., Zheng Y., Lei X., Tang M., Wang H., Song F. (2017). Chemical Composition, Antibacterial and Antioxidant Activities of Hydrosols from Different Parts of *Areca catechu* L. and *Cocos nucifera* L.. Ind. Crops Prod..

[12] Aćimović M., Tešević V., Smiljanić K., Cvetković M., Stanković J., Kiprovski B., Sikora V. (2020). Hydrolates: By-Products of Essential Oil Distillation: Chemical Composition, Biological Activity and Potential Uses. Adv. Technol..

[13] Adams R.P. (2017). Identification of Essential Oil Components by Gas Chromatography/Mass Spectrometry.

[14] Paparella A., Shaltiel-harpaza L., Ibdah M. (2021). Β-Ionone: Its Occurrence and Biological Function and Metabolic Engineering. Plants.

[15] Cid-Pérez T.S., Ávila-Sosa R., Ochoa-Velasco C.E., Rivera-Chavira B.E., Nevárez-Moorillón G.V. (2019). Antioxidant and Antimicrobial Activity of Mexican Oregano (*Poliomintha longiflora*) Essential Oil, Hydrosol and Extracts Fromwaste Solid Residues. Plants.

[16] Mendes C.E., Flach A., da Costa L.A.M.A., Denardin R.B.N., de Moura N.F. (2014). Chemical Composition and Multivariate Analysis of the Volatile Oil of *Dalbergia frutescens* (Vell.) Britton (Fabaceae). J. Braz. Chem. Soc..

[17] Sugier D., Olesińska K., Sugier P., Wójcik M. (2019). Chemical Composition of Essential Oil from Flower Heads of Arnica Chamissonis Less. Under a Nitrogen Impact. Molecules.

[18] Zhou Y., Cao H.B., Li W.J., Zhao L. (2018). The CXCL12 (SDF-1)/CXCR4 Chemokine Axis: Oncogenic Properties, Molecular Targeting, and Synthetic and Natural Product CXCR4 Inhibitors for Cancer Therapy. Chin. J. Nat. Med..

[19] Huang X.Q., Li R., Fu J., Dudareva N. (2022). A Peroxisomal Heterodimeric Enzyme Is Involved in Benzaldehyde Synthesis in Plants. Nat. Commun..

[20] Neto L.J.D.L., Ramos A.G.B., de Freitas T.S., Barbosa C.R.D.S., de Sousa Júnior D.L., Siyadatpanah A., Nejat M., Wilairatana P., Coutinho H.D.M., da Cunha F.A.B. (2021). Evaluation of Benzaldehyde as an Antibiotic Modulator and Its Toxic Effect against Drosophila Melanogaster. Molecules.

[21] Rubab M., Chelliah R., Saravanakumar K., Barathikannan K., Wei S., Kim J.R., Yoo D., Wang M.H., Oh D.H. (2020). Bioactive Potential of 2-Methoxy-4-Vinylphenol and Benzofuran from *Brassica oleracea* L. Var. Capitate f, Rubra (Red Cabbage) on Oxidative and Microbiological Stability of Beef Meat. Foods.

[22] Jeong J.B., Hong S.C., Jeong H.J., Koo J.S. (2011). Anti-Inflammatory Effect of 2-Methoxy-4-Vinylphenol via the Suppression of NF-ΚB and MAPK Activation, and Acetylation of Histone H3. Arch. Pharm. Res..

[23] Chavan M.J., Wakte P.S., Shinde D.B. (2010). Analgesic and Anti-Inflammatory Activity of Caryophyllene Oxide from *Annona squamosa* L. Bark. Phytomedicine.

[24] Su Y.C., Hsu K.P., Ho C.L. (2018). Composition, In Vitro Anti-Mildew Fungal Activities of the Heartwood Essential Oil of *Chamaecyparis formosensis* from Taiwan. Nat. Prod. Commun..

[25] Viljoen G.P.P.K., Alvaro M.V. (2014). Linalool—A Review of a Biologically Active Compound of Commercial Importance. Nat. Prod. Commun..

[26] Lucchesi M.E., Chemat F., Smadja J. (2004). Solvent-Free Microwave Extraction of Essential Oil from Aromatic Herbs: Comparison with Conventional Hydro-Distillation. J. Chromatogr. A.

[27] Paolini J., Leandri C., Desjobert J.M., Barboni T., Costa J. (2008). Comparison of Liquid-Liquid Extraction with Headspace Methods for the Characterization of Volatile Fractions of Commercial Hydrolats from Typically Mediterranean Species. J. Chromatogr. A.

[28] Moradi S., Fazlali A., Hamedi H. (2018). Microwave-Assisted Hydro-Distillation of Essential Oil from Rosemary: Comparison with Traditional Distillation. Avicenna J. Med. Biotechnol..

[29] Sourmaghi M.H.S., Kiaee G., Golfakhrabadi F., Jamalifar H., Khanavi M. (2015). Comparison of Essential Oil Composition and Antimicrobial Activity of *Coriandrum sativum* L. Extracted by Hydrodistillation and Microwave-Assisted Hydrodistillation. J. Food Sci. Technol..

[30] Popović M., Jukić Špika M., Veršić Bratinčević M., Ninčević T., Matešković A., Mandušić M., Rošin J., Nazlić M., Dunkić V., Vitanović E. (2021). Essential Oil Volatile Fingerprint Differentiates Croatian Cv. Oblica from Other *Olea europaea* L. Cultivars. Molecules.

[31] Craft J.D., Satyal P., Setzer W.N. (2017). The Chemotaxonomy of Common Sage (*Salvia officinalis*) Based on the Volatile Constituents. Medicines.

[32] Wang Y., Li X., Jiang Q., Sun H., Jiang J., Chen S., Guan Z., Fang W., Chen F. (2018). GC-MS Analysis of the Volatile Constituents in the Leaves of 14 Compositae Plants. Molecules.

[33] Giuliani C., Lazzaro L., Calamassi R., Calamai L., Romoli R., Fico G., Foggi B., Mariotti Lippi M. (2016). A Volatolomic Approach for Studying Plant Variability: The Case of Selected *Helichrysum* Species (Asteraceae). Phytochemistry.

[34] Mocan A., Vodnar D.C., Vlase L., Crișan O., Gheldiu A.M., Crișan G. (2015). Phytochemical Characterization of *Veronica officinalis* L., *V. teucrium* L. and *V. orchidea* Crantz from Romania and Their Antioxidant and Antimicrobial Properties. Int. J. Mol. Sci..

[35] Valyova M., Hadjimitova V., Stoyanov S., Ganeva Y., Petkov I. (2008). Free Radical Scavenging Activity of Extracts from Bulgarian *Veronica officinalis* L. and GC-MS Analysis of Ethanol Extract. Internet J. Aesthetic Antiaging Med..

[36] Harput U.S., Saracoglu I., Genc Y. (2009). Comparative Bioactivity Studies on Four *Veronica* Species. Fabad J. Pharm. Sci..

[37] Sharifi-Rad J., Sharifi-Rad M., Salehi B., Iriti M., Roointan A., Mnayer D., Soltani-Nejad A., Afshari A. (2018). In Vitro and in Vivo Assessment of Free Radical Scavenging and Antioxidant Activities of *Veronica persica* Poir. Cell. Mol. Biol..

[38] Dunkić V., Kosalec I., Kosir I., Potočnik T., Cerenak A., Koncic M., Vitali D., Muller I., Kopricanec M., Bezić N. (2015). Antioxidant and Antimicrobial Properties of *Veronica spicata* L. (Plantaginaceae). Curr. Drug. Targets.

[39] Živković J., Ćebović T., Maksimović Z. (2012). In Vivo and in Vitro Antioxidant Effects of Three *Veronica* Species. Cent. Eur. J. Biol..

[40] Grubešić R.J., Nazlić M., Miletić T., Vuko E., Vuletić N., Ljubenkov I., Dunkić V. (2021). Antioxidant Capacity of Free Volatile Compounds from *Olea europaea* l. Cv. Oblica Leaves Depending on the Vegetation Stage. Antioxidants.

[41] NIST Chemistry WebBook. https://webbook.nist.gov/.

[42] Ertas A., Boga M., Kizil M., Ceken B., Goren A.C., Hasimi N., Demirci S., Topcu G., Kolak U. (2015). Chemical Profile and Biological Activities of *Veronica Thymoides* Subsp. Pseudocinerea. Pharm. Biol..

[43] Li F. (2002). Analysis of Chemical Constituents of Essential Oil in *Veronica linariifolia* by Gas Chromatography-Mass Spectrometry. Chin. J. Anal. Chem..

[44] Nazlić M., Kremer D., Grubešić R.J., Soldo B., Vuko E., Stabentheiner E., Ballian D., Bogunić F., Dunkić V. (2020). Endemic *Veronica saturejoides* Vis. ssp. Saturejoides–Chemical Composition and Antioxidant Activity of Free Volatile Compounds. Plants.

[45] Fredotović Ž., Šprung M., Soldo B., Ljubenkov I., Budić-Leto I., Bilušić T., Cikeš-Čulić V., Puizina J. (2017). Chemical Composition and Biological Activity of *Allium cepa* L. and *Allium* × *Cornutum* (Clementi Ex Visiani 1842) Methanolic Extracts. Molecules.

[46] Mensor L.L., Menezes F.S., Leitão G.G., Reis A.S., dos Santos T.C., Coube C.S., Leitão S.G. (2001). Screening of Brazilian Plant Extracts for Antioxidant Activity by the Use of DPPH Free Radical Method. Phytother. Res..

[47] Payet B., Sing A.S.C., Smadja J. (2005). Assessment of Antioxidant Activity of Cane Brown Sugars by ABTS and DPPH Radical Scavenging Assays: Determination of Their Polyphenolic and Volatile Constituents. J. Agric. Food Chem..

[48] Yen G.-C., Duh P.-D. (1994). Scavenging Effect of Methanolic Extracts of Peanuts Hulls on Free-Radical and Active-Oxygen Species. J. Agric. Food Chem..

